# Iodine Biofortification of Apples and Pears in an Orchard Using Foliar Sprays of Different Composition

**DOI:** 10.3389/fpls.2021.638671

**Published:** 2021-02-24

**Authors:** Christoph Budke, Werner Dierend, Hans-Georg Schön, Katja Hora, Karl Hermann Mühling, Diemo Daum

**Affiliations:** ^1^Faculty of Agricultural Sciences and Landscape Architecture, Osnabrück University of Applied Sciences, Osnabrück, Germany; ^2^SQM International N.V, Antwerpen, Belgium; ^3^Faculty of Agricultural and Nutritional Sciences, Institute of Plant Nutrition and Soil Science, Kiel University, Kiel, Germany

**Keywords:** pome fruit, agronomic biofortification, foliar fertilization, iodide, iodate, selenium, potassium nitrate, total soluble solids

## Abstract

Many people across the world suffer from iodine (I) deficiency and related diseases. The I content in plant-based foods is particularly low, but can be enhanced by agronomic biofortification. Therefore, in this study two field experiments were conducted under orchard conditions to assess the potential of I biofortification of apples and pears by foliar fertilization. Fruit trees were sprayed at various times during the growing season with solutions containing I in different concentrations and forms. In addition, tests were carried out to establish whether the effect of I sprays can be improved by co-application of potassium nitrate (KNO_3_) and sodium selenate (Na_2_SeO_4_). Iodine accumulation in apple and pear fruits was dose-dependent, with a stronger response to potassium iodide (KI) than potassium iodate (KIO_3_). In freshly harvested apple and pear fruits, 51% and 75% of the biofortified iodine was localized in the fruit peel, respectively. The remaining I was translocated into the fruit flesh, with a maximum of 3% reaching the core. Washing apples and pears with running deionized water reduced their I content by 14%. To achieve the targeted accumulation level of 50–100 μg I per 100 g fresh mass in washed and unpeeled fruits, foliar fertilization of 1.5 kg I per hectare and meter canopy height was required when KIO_3_ was applied. The addition of KNO_3_ and Na_2_SeO_4_ to I-containing spray solutions did not affect the I content in fruits. However, the application of KNO_3_ increased the total soluble solids content of the fruits by up to 1.0 °Brix compared to the control, and Na_2_SeO_4_ in the spray solution increased the fruit selenium (Se) content. Iodine sprays caused leaf necrosis, but without affecting the development and marketing quality of the fruits. Even after three months of cold storage, no adverse effects of I fertilization on general fruit characteristics were observed, however, I content of apples decreased by 20%.

## Introduction

Iodine is an integral component of thyroid hormones, which control various metabolic processes in the human body. Globally, around two billion people are insufficiently supplied with this essential trace element (Andersson et al., 2012). The associated health disorders range from mild, unspecific symptoms such as listlessness to severe neurological developmental disorders. Iodine deficiency is considered to be the most common single cause of preventable brain damage and intellectual disability in children worldwide (Benoist et al., 2009; Redman et al., 2016). Even a mild to moderate I deficiency during pregnancy and in the first years of life can lead to children not being able to fully exploit their cognitive development potential (Velasco et al., 2018; Bath, 2019). The problem of I deficiency exists in both developing and industrialized countries. In Europe, about 44% of the population is inadequately supplied with I, despite its wealth and its high standards of health care (Zimmermann, 2017). The widespread occurrence of I deficiency is due to the fact that the native I content in food is usually very low. Food crops such as fruits, vegetables and cereals usually contain no more than about 1.0 μg of I per 100 g of fresh mass, since soils are low in phytoavailable I, and therefore the absorption of this trace element by plants is quite limited (Fuge, 2013; Milagres et al., 2020).

An option for increasing the I content of food crops is therefore to fertilize the soil with I-containing salts. Various studies show that this measure actually has an effect, but requires relatively high amounts of I fertilizer (Ren et al., 2008; Weng et al., 2014). This is due to the relatively rapid fixation of I in the soil when applied as iodide (I^–^) or iodate (IO_3_^–^). In addition, these inorganic I forms can be converted by soil microorganisms into gaseous compounds such as methyl iodide, which are emitted into the atmosphere (Ashworth, 2009; Shetaya et al., 2012; Fuge, 2013). While leafy and root vegetables respond relatively well to I soil fertilization, only little I reaches the edible plant parts of fruit vegetables and cereals using this method (Hong et al., 2008; Cakmak et al., 2017). Compared to soil fertilization, foliar applications proved to be much more efficient. For example, it was possible to biofortify lettuce adequately with I if the plants were sprayed with 0.5 kg I ha^–1^ one week before harvest. With soil drenches, a 15-fold higher I fertilizer quantity was required for the same I enrichment in this leafy vegetable (Lawson et al., 2015). Also in experiments carried out with strawberries and cereals, foliar sprays proved to be superior to soil fertilization in order to increase the I content in the fruits and grains, respectively (Cakmak et al., 2017; Budke et al., 2020b).

In this study apples and pears were selected as target crops for I biofortification via foliar sprays. These fruits have several characteristics that make them particularly suitable for improving the dietary I intake in I deficiency areas. First of all, apple and pear are among the ten most important fruit species in the world with a production of 86 million tons and 24 million tons, respectively (FAO, 2020). Fruits can be stored for a long time – pears for a few months, while apples from domestic production can be offered in food stores throughout the year. Pome fruits are usually eaten with the peel, while other fruits that are regularly consumed in larger quantities, such as bananas and citrus fruits, are peeled. This is important because a previous study on apples showed that more than half of the foliar-sprayed I is localized in the fruit peel. Nevertheless, I in the peel is hardly affected by washing of the fruit under running water – this reduced the fruit I content by only 8% (Budke et al., 2020a). Thus, when fresh pome fruits are consumed, most of the biofortified I usually becomes nutritionally effective. In contrast, processed foods, such as potatoes, vegetables, and cereals, may experience significant I losses through cooking, peeling, or extraction compared to harvested produce. Even then, however, enough I remains in the biofortified plant-based foods to substantially increase dietary supply of this micronutrient (Caffagni et al., 2012; Gonnella et al., 2019; Cakmak et al., 2020). Loss of I from iodized table salt during food preparation can be much higher. When cooking vegetables or potatoes with iodized table salt, only very little amounts of the I dissolved in the cooking water enters the prepared food (Comandini et al., 2013; Weng et al., 2014).

The inorganic I^–^ and IO_3_^–^ forms, which are mainly used for the biofortification of food plants, are characterized by a high bioavailability (> 95%) in the human organism (Hou, 2009). After the incorporation of I into plant tissue, it is mainly present in the cytoplasm, and to a smaller extent in the cell wall or the organelles (Weng et al., 2014). Iodine can be incorporated into various organic compounds such as proteins, lipids, polysaccharides and polyphenols (Millard, 1988; Hou, 2009), and occurs naturally in the form of triiodothyronines or other iodo-tyrosins in lettuce and tomato plants even if they are not receiving exogenous I (Halka et al., 2019; Sularz et al., 2020). Recently, in a study on proteomics in *Arabidopsis thaliana* (L.), I has been found to be organified in many important regulatory proteins of the plant, pointing to a nutritional role of I for plants at concentrations which are generally much lower than the I levels applied for purpose of biofortification (Kiferle et al., 2020). So far, little is known about which of these organic I species play a major role in I-fertilized plants. Nevertheless, several studies conducted in vitro and as clinical trials indicate that biofortified I remains largely bioavailable in plant foods (Tonacchera et al., 2013; Li et al., 2018; Cakmak et al., 2020).

Previous work showed that it is possible to biofortify apples with I via foliar fertilization in an order of magnitude appropriate for improving the dietary I intake. However, this required that the applied KI-containing solution was supplied directly to the fruits. No significant translocation of I from the leaves to the fruits was observed, although the I content in the leaves rose up to over 2,000 μg (100 g FM)^–1^ as a result of the treatment. Thus it was concluded that leaf-absorbed I in apple trees is hardly translocated via the phloem (Budke et al., 2020a). The aforementioned study was performed on apple trees cultivated in a plastic tunnel. The trees were protected from precipitation and temporarily exposed to a microclimate with higher humidity. These conditions may have favored the absorption of the sprayed I into the fruit. Therefore, the present study was designed to evaluate the efficacy of I biofortification under field conditions in an apple and pear orchard.

Regarding the effect of the I form – I^–^ versus IO_3_^–^ – on I accumulation in plants, different results are reported in the literature. In some cases, foliar-applied I^–^ proved to be more easily absorbable, while in other experiments no consistent differences between the two I forms could be observed (Lawson et al., 2015; Lawson et al., 2016; Cakmak et al., 2017). At higher fertilization rates, however, IO_3_^–^ is generally better tolerated by plants than I^–^ (Dávila-Rangel et al., 2019). Therefore, we examined in our field experiments how treatments with both I species affected the development and external appearance of leaves and fruits.

Various additives can be used to improve the effect of foliar fertilization. Surfactants contribute to improve wetting of the sprayed above-ground plant parts (Fernández et al., 2013). They are particularly important for the treatment of pome fruit, as the fruits form a epicuticular wax layer during their development which is much thicker than that of leaves (Fernandes et al., 1964). The hydrophobic coatings impair the penetration of ionic solutes into the fruit. In I fertilization experiments with wheat, apart from a wetting agent, the addition of KNO_3_ to the spray solution had a positive effect on the absorption and translocation of the trace element in the plant. The I content in grains was 1.5–2.3 times higher when I was sprayed with a wetting agent or KNO_3_ than when I was applied alone (Cakmak et al., 2017). In the present study all foliar sprays were supplied with a surfactant additionally. Furthermore, the effect of co-fertilization of KNO_3_ on I accumulation in apples and pears was investigated.

In addition to I, Se plays an important role in normal thyroid function (Schomburg and Köhrle, 2008). In many European countries and other regions of the world, the native Se content in plant-based foods is very low and therefore an insufficient dietary intake of Se is also widespread (Rayman, 2008; Peters et al., 2016). Again, as with I, the original reason for this is the low phytoavailability of the trace element in soils (Poòaviè and Scheib, 2014; dos Reis et al., 2017). Simultaneous biofortification of food crops with I and Se is therefore considered to be a useful strategy for the prevention of thyroid diseases (Lyons, 2018).

Biofortified fruit can be marketed with nutritional claims such as “rich in iodine”. The willingness of customers to buy such products is even greater when other quality characteristics such as the taste of the fruit are also appealing (Wortmann et al., 2018). The sugar content affects the degree of sweetness and thus the taste of the fruits (Aprea et al., 2017; Charles et al., 2017). Both I and Se are known to influence the allocation of photoassimilates in plants. Studies on strawberries have shown that, depending on amount, form and application technique, I fertilization can have beneficial and inhibitory effects on the accumulation of soluble solids in the fruits, which are mainly composed of sugars (Li et al., 2017; Budke et al., 2020b). Spraying of pear trees with sodium selenate (Na_2_SeO_4_) resulted in a significant increase in the total soluble solids content of the fruits (Pezzarossa et al., 2012). In addition, foliar applications with KNO_3_ can enhance fructose and sucrose content, as was observed in ‘Kousui’ Japanese pears (*Pyrus pyrifola*) (Shen et al., 2016). Therefore, we also included a combination treatment consisting of I with Na_2_SeO_4_ and KNO_3_ in our field experiments.

During storage of pome fruits, physiological processes can affect the quality of the fruit and its nutrient composition in many aspects (Thompson et al., 2018; Brizzolara et al., 2020). Therefore, it is important to understand if there are storage-related changes in I and Se contents. Additionally, the distribution and translocation of the trace elements within biofortified fruits (fruit peel, fruit flesh and fruit core) were studied from harvest through storage.

Overall, the aim of this study was to investigate various aspects of I biofortification of apples and pears by different foliar spray treatments during cultivation in an orchard, relevant for an implementation of this approach in fruit growing practice. We tested the hypothesis that by this method these pome fruits can be enriched with I at a level sufficient for improvement of human I nutrition.

## Materials and Methods

### Plant Material and Growing Conditions

**FIGURE 1:**
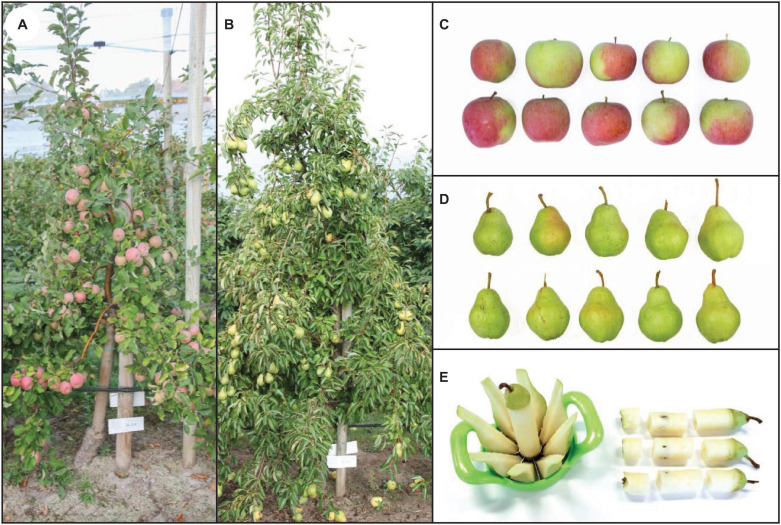
Examples of fruit trees included in the second field experiment and fruit appearance shortly after the harvest: Apple tree cv. ‘Fuji’ **(A)**, pear tree cv. ‘Williams Christ’ **(B)**. Selection of 10 harvested apple **(C)** and pear fruits **(D)** from treatment no. 5 consisting of a combined foliar spray with KNO_3_, KIO_3_ and Na_2_SeO_4_ which did not negatively affect external fruit characteristics. Partitioning of fruits for further preparation and analysis steps **(E)**.

Field experiments were conducted on two sites in an orchard of the horticultural trial station of the Osnabrück University of Applied Sciences (site 1: 52°18′23.5″N 8°02′23.7″E; site 2: 52°18′39.1″N 8°01′42.3″E). Both neighboring locations (distance approx. 1.2 km as the crow flies) were characterized by a plaggen soil of loamy-sand texture. Soil analyses in representative samples were conducted in 2012 (for site 1) and 2017 (for site 2). The results are shown in Table 1. The first field experiment was carried out in 2013 on site 1 and included apple trees (*Malus domestica*) of the variety ‘Jonagold’ and pear trees (*Pyrus communis*) of the variety ‘Alexander Lucas’. The second field experiment took place on site 2 in 2018 with apple trees of the variety ‘Fuji’ and pear trees of the variety ‘Williams Christ’. Here, the soil was fertilized with 90 kg K_2_O ha^–1^ in spring. The planting distances of the trees were 3.25 m × 1.0 m for the apple trees and 3.25 m × 1.5 m for the pear trees. This corresponds to a total number of 3,076 apple trees and 2,051 pear trees per hectare. The trees had an average height of 2.5 – 3.0 m and were grown in spindle form with a dominant trunk (Figures 1A,B).> The average air temperature, precipitation quantity and number of rainy days were 14.2 °C, 392.5 mm and 88 days, respectively, between April and October 2013. For the corresponding period in 2018, the values were 16.1°C, 268.6 mm and 70 days, respectively.

**TABLE 1:** Results of the soil analyses from the experimental sites.

**Soil parameter**	**First field trial (site 1)**		**Second field trial (site 2)**	
**Topsoil (0 - 30 cm)**	**Subsoil (30 - 60 cm)**	**Topsoil (0 - 30 cm)**	**Subsoil (30 - 60 cm)**
Phosphorus (CAL)*	D	D	C	C
Potassium (CAL)*	D	C	C	C
Magnesium (CaCl_2_)	E	D	C	C
pH (CaCl_2_)	5.6	5.9	5.0	5.1
Humus content (%)	2.3	1.7	1.8	1.1
CaCl_2_-extractable iodine (mg kg^–1^)	< 0.025	<0.025	< 0.025	<0.025
Aqua regia-extractable selenium (mg kg^–1^)	–	–	0.21	0.19

Capital letters indicate nutrient supply class (A, low; B, slightly low; C, optimal; D, slightly high; E, high) according to the Association of German Agricultural Analytic and Research Institutes – VDLUFA (Kießling and Hoffmann, 2016). *CAL extraction solution contains 0.05 *M* calcium lactate, 0.05 *M* calcium acetate, and 0.3 *M* acetic acid per liter and is buffered at pH 4.1.

### Foliar Spray Treatments

The first field experiment was aimed at determining the influence of the I fertilizer dose and form in foliar sprays on I accumulation in apples and pears. Here, potassium iodide (KI) and potassium iodate (KIO_3_) were applied as pure salts (VWR International GmbH, Bruchsal, Germany) in three different application rates each (Table 2). In the second field experiment, the effect of I fertilization in combination with further foliar spray treatments was investigated. The following fertilizers were used: KIO_3_ as Speedfol^®^ Iodine SP and KNO_3_ as Ultrasol^®^ K Plus, both as powder (SQM EUROPE N.V., Antwerp, Belgium) as well as sodium selenate (Na_2_SeO_4_), analytical-grade quality (Thermo Fisher Scientific, Kandel, Germany). Detailed information on the spray solutions are provided in Table 2. For all foliar sprays the surfactant Break-Thru^®^ S 240 (AlzChem AG, Trostberg, Germany) was used in a concentration of 0.02% v/v.

**TABLE 2:** Composition of the spray solutions used in the field experiments.

**First field trial**			**Second field trial**		
**Treatment**	**Total foliar application dose [kg (ha ⋅ m CH)^–1^] and chemical form**		**Treatment**	**Total foliar application dose [kg (ha ⋅ m CH)^–1^] and chemical form**	
	
1	0	Control	1	0	Control
			2	20	KNO_3_
	
2	0.25	KI	3	1.5	KIO_3_
	
3	1.0	KI	4	1.5	KIO_3_
4	2.5	KI		20	KNO_3_
	
5	0.25	KIO_3_	5	1.5	KIO_3_
6	1.0	KIO_3_		0.05	Na_2_SeO_4_
7	2.5	KIO_3_		20	KNO_3_

*Applied doses are indicated per hectare and meter canopy height (CH) with the adjuvant Break-Thru^®^ S 240 (0.02% v/v) at a water amount of 1,000 L (ha ⋅ m CH)^–1^.*

All foliar treatments were supplied to the entire canopy of fruit trees, i.e. leaves and fruits. In the first field experiment the spray solutions were applied once two weeks before harvest of the apples or pears using a handheld sprayer (Easy Sprayer Plus, Lehnartz GmbH, Remscheid, Germany). In the second field experiment the treatments took place with a backpack sprayer (REB 15 AZ2, Birchmeier Sprühtechnik AG, Stetten, Switzerland) and were split into several dates. For apples, two applications were carried out and for pears three (Table 3). The water application rate was 1,000 L (ha ⋅ m CH)^–1^ (CH = canopy height) in each case. The water application rates chosen ensured that the spray solutions did not run off the plant surface. The treatments were always carried out in the morning hours with no wind and in dry weather conditions.

**TABLE 3:** Splitting of the total foliar application dose, application dates and harvest dates in the conducted field experiments.

**First field trial**			**Second field trial**		
	**Fruit species**			**Fruit species**	
	**Apple**	**Pear**		**Apple**	**Pear**
Number of applications	1	1	Number of applications	2	3
Treatment	Sep. 15	Sep. 13	1^st^ treatment	Jul. 26	Jun. 19
			2^nd^ treatment	Aug. 31	Jul. 23
			3^rd^ treatment	-	Aug. 6
Harvest date	Sep. 30	Sep. 24	Harvest date	Oct. 8	Aug. 20
			End of fruit storage	Jan. 10	Nov. 21

### Data Collection, Sampling and Sample Preparation

The trees were checked for leaf and fruit damage four times during the test period and were rated accordingly (1 = no damage, 9 = very severe damage). Only fruits that were positioned in the outer part of the tree were included in the sampling for analytical investigations to ensure that they were directly wetted by the spray solution. 20 fruits per tree were harvested and the individual fruit weight was determined. In 2018 half of the fruits were stored for three months at 2°C (Table 3). After harvest and storage, the external appearance of the fruits was visually evaluated and photographically documented (Figures 1C,D). In the second field experiment leaf samples were also taken for analytical purposes from the apple trees at harvest time. For this purpose about 20 – 30 leaves per tree were collected near the sampled fruits.

During fruit processing the fruits were initially divided vertically into eight equal segments and the core cylinder (Figure 1E). Two opposite fruit segments were then processed unwashed, washed or peeled. The washing was carried out under running deionised water. A fine peeler was used for peeling the fruit segments. The middle part of the core cylinder was used for the analytical examination and the upper and lower parts were discarded. The fruit samples were dried at 60°C in a forced air oven (TUH 75/100, Heraeus Holding GmbH, Hanau, Germany) until the weight was constant. Using an ultracentrifugal mill (ZM 200, RETSCH GmbH, Haan, Germany), the samples were then ground at 14,000 rpm to a particle size of ≤ 0.5 mm. Until analysis the sample material obtained in this way was stored in sealed plastic beakers and dried again shortly before chemical digestion.

### Analyses of Iodine and Selenium in Plant Samples

The I determination was performed according to the method DIN EN 15111 (2007). Briefly, 1 g of dried plant substance was used and chemically digested with 25% tetramethylammonium hydroxide solution (TMAH). To ensure the quality of the analysis, certified reference material (ERM-BB422 fish muscle and NIST-1849a infant/adult nutritional milk powder) was used. Another reference material was apple powder from our own experiments, which had been previously analyzed in an external laboratory accredited for I analysis in food (LUFA Nord-West, Hameln, Germany). The I determination was performed by inductively coupled plasma mass spectrometry (ICP-MS, Agilent 7700, Agilent Technologies Inc., Santa Clara, CA, United States).

Selenium was determined according to the method DIN EN 13805 (2014). For this purpose, 0.5 g of the ground plant material was digested by microwave pressure digestion in quartz glass vessels with 65% nitric acid at a temperature of 190°C and under pressure. The digestion solution was analyzed by graphite furnace atomic absorption spectrometry (GF-AAS, Thermo Scientific – SOLAAR M Series AA Spectrometer, Thermo Fisher Scientific Inc., Waltham, MA, United States). For quality control purposes, the same certified reference materials were used as for I analysis. Again, comparative tests were performed in an external laboratory accredited for Se determination (LUFA Nord-West, Hameln, Germany). Samples with Se concentrations below 2.5 μg L^–1^ were analyzed by using the hydride technique in accordance with the method DIN 38405-23 (1994).

The I and Se content of the fruit peel was calculated from the difference between washed and peeled fruit segments. In the second field experiment the I and Se contents were also determined in unwashed and washed apple leaves, once in the control (treatment 1) and once in the variants fertilized with KIO_3_ (treatment 3) and KIO_3_ + Na_2_SeO_4_ + KNO_3_ (treatment 5).

### Measurement of Total Soluble Solids Content

Two segments per fruit were used to determine the total soluble solids content. The sample material was pureed and then filtered. The resulting juice was analyzed with a digital refractometer (PAL-1, ATAGO CO., Ltd., Tokyo, Japan).

### Trial Set-Up and Statistical Procedures

The field experiments were designed as randomized block experiments with usually four replications. The experiment with apple trees in 2018 included six replications. Each treatment was represented by one tree per block. The selection of the trees was based on a homogeneous structure and fruit number. To avoid edge effects, the treated trees were separated from each other by at least one untreated tree. In addition, plastic foil barriers were used to isolate each tree during the spraying process to prevent contamination by drift.

The results obtained in the fruit analyses were subjected to one-way or two-way ANOVA and, if needed, logarithmized to meet assumptions of normality and homogeneity of variances. Multiple mean value comparisons were made using the Tukey-HSD test and the LSD test. The program IBM SPSS^®^ Statistics, version 26 (IBM Deutschland GmbH, Ehningen, Germany), was used for statistical data evaluation.

## Results

### Iodine Content of Fruits and Leaves

**FIGURE 2:**
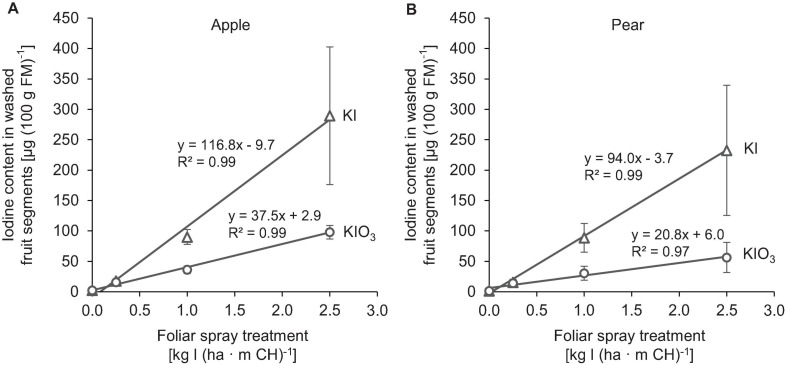
Iodine content in washed fruit segments of apples cv. ‘Jonagold’ **(A)** and pears cv. ‘Alexander Lucas’ **(B)** at harvest time as affected by the dose and form of iodine foliar sprays in the first field experiment. Means ± standard deviation (*n* = 4).

**FIGURE 3:**
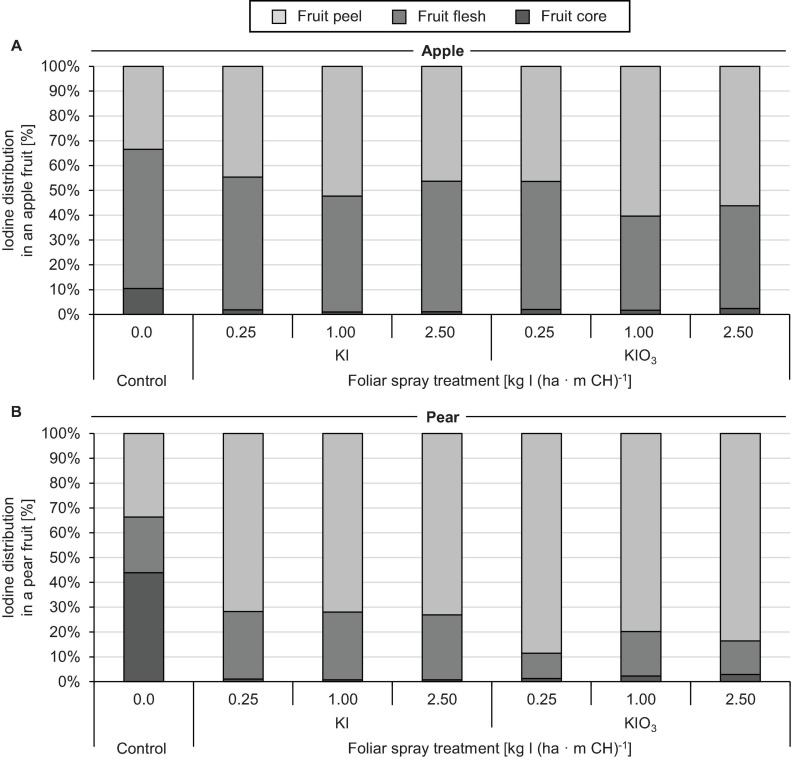
Iodine distribution in washed apples cv. ‘Jonagold’ **(A)** and pears cv. ‘Alexander Lucas’ **(B)** at harvest time as affected by the dose and form of iodine foliar sprays in the first field experiment.

The native I content of apples and pears was 1.5 μg (100 g FM)^–1^ and 1.0 μg (100 g FM)^–1^, respectively. Foliar sprays with I-containing solutions significantly increased the I content of the fruits. In the first field experiment, a single treatment with 0.25 – 2.50 kg I (ha ⋅ m CH)^–1^, carried out two weeks before harvest, led to an increase in the I content in washed fruit segments from 15.7 μg (100 g FM)^–1^ up to more than 200 μg (100 g FM)^–1^ (Figure 2). The mean dry matter (DM) content was 16% for apple and pear. The aforementioned values thus correspond 0.9 to > 12.5 mg I (kg DM)^–1^. There was a close linear relationship between the I fertilization level and the I enrichment of the fruits. Further statistical analysis shows that the mean I content determined for the different I doses and forms were predominantly significantly different (Supplementary Tables 1, 2). The application of I^–^ resulted, averaged over both fruit species, in an I content that was 2.5 times higher than a corresponding supply of IO_3_^–^. However, the I enrichment of I^–^-treated fruits, especially at the highest supply rate, varied much more than when using the oxidized I form. Figure 2 also shows that apples tended to accumulate more I per unit of weight than pears. These differences in I content were related to the different individual fruit weights. Pears harvested in this trial were 19% heavier than a single apple fruit. The total amount of I per fruit contained in apples and pears was similar with the same I form and dose and reached a maximum of 508 μg/fruit for apple and 467 μg/fruit for pear at the highest I^–^ supply rate (Table 4). In I-sprayed apples, 51, 47, and 2% of the I was localized in the fruit peel, the fruit flesh and the fruit core, respectively. For pears the corresponding values were 78, 20, and 2%, respectively (Figure 3). Compared to KIO_3_, the supply of KI favored the translocation of the I into the fruit flesh. Washing the fruits in running deionised water reduced the I content in the first field experiment by 14% for apples and 16% for pears.

**TABLE 4:** Iodine amount in a whole washed fruit including core and individual fruit weight of apples and pears from the first and the second field experiment as affected by the spray solution.

				**Apple**				**Pear**			
**Treatment^1)^**				**Iodine amount per fruit [μg]**		**Individual fruit weight [g]**		**Iodine amount per fruit [μg]**		**Individual fruit weight [g]**	
		
First field trial	1	Control	0	4.7 ± 0.2	a	206.8 ± 13.6	a	2.7 ± 1.5	a	230.9 ± 26.8	a
			
	2	KI	0.25	31.3 ± 4.7	b	195.2 ± 22.1	a	30.9 ± 10.2	b	238.8 ± 5.0	a
	3		1.0	170.9 ± 23.5	d	210.9 ± 5.8	a	172.4 ± 45.9	d	232.0 ± 37.2	a
			*1.5^2)^*	*294.1*				*274.6*			
	4		2.5	508.0 ± 198.7	e	194.0 ± 13.2	a	467.4 ± 215.8	e	239.7 ± 33.7	a
			
	5	KIO_3_	0.25	28.8 ± 8.2	b	199.4 ± 11.2	a	29.4 ± 12.1	b	246.2 ± 63.8	a
	6		1.0	65.2 ± 10.7	c	196.2 ± 8.6	a	59.3 ± 22.5	bc	227.5 ± 33.3	a
			*1.5^2)^*	*105.5*				*71.7*			
	7		2.5	173.9 ± 22.1	d	191.2 ± 28.8	a	107.3 ± 47.9	cd	219.6 ± 28.4	a

Second field trial	1	Control	0	1.6 ± 0.4	a	211.2 ± 39.7	a	1.8 ± 1.0	a	166.8 ± 18.0	a
			
	2	KNO_3_	0	1.6 ± 0.7	a	214.2 ± 31.1	a	1.9 ± 0.7	a	154.5 ± 30.4	a
	3	KIO_3_	1.5	102.7 ± 10.8	b	209.0 ± 21.9	a	79.9 ± 6.2	b	157.1 ± 11.6	a
	4	KIO_3_ + KNO_3_	1.5	89.7 ± 14.4	b	204.5 ± 37.8	a	87.8 ± 11.2	b	150.7 ± 7.1	a
	5	KIO_3_ + Na_2_SeO_4_ + KNO_3_	1.5	84.0 ± 13.5	b	194.2 ± 31.2	a	85.7 ± 13.8	b	143.0 ± 18.1	a

*Means ± standard deviation (*n* = 4, except ‘Fuji’ apples second field trial *n* = 6). Means with same letters in one column for one field trial do not differ according to Tukey-HSD test at α = 0.05. ^1)^ Iodine application rate expressed in kg (ha ⋅ m CH)^–1^. ^2)^ Values calculated for comparison purposes are based on the regression equations indicated in Figure 2.*

**FIGURE 4:**
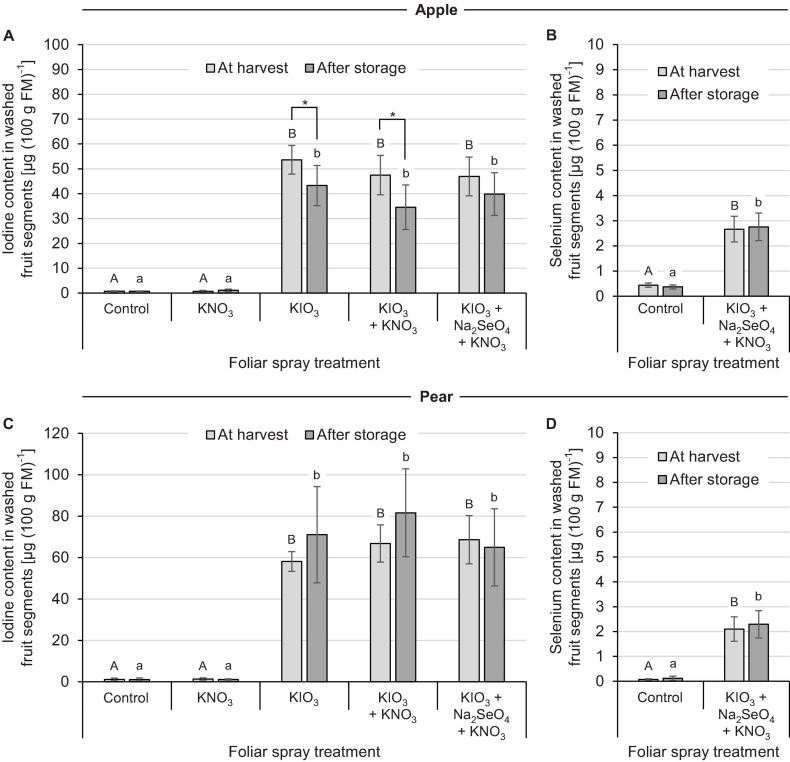
Iodine and selenium content in washed fruit segments of apples cv. ‘Fuji’ **(A,B)** and pears cv. ‘Williams Christ’ **(C,D)** in the second field experiment as affected by different foliar spray treatments and fruit storage at 2°C for a period of three months. Means ± standard deviation (apple *n* = 6, pear *n* = 4). Means not sharing a letter in one chart or indicated by an asterisk are significantly different according to Tukey-HSD test at α = 0.05.

In the second field experiment the effect of I spraying in combination with further foliar fertilization treatments was investigated. In contrast to the previous experiment, only KIO_3_ with a uniform application rate of 1.5 kg I (ha ⋅ m CH)^–1^ was used. Furthermore, the applications were split into two dates for apple and three dates for pear. The addition of KNO_3_ and Na_2_SeO_4_ to the I spray solution had no clear influence on the I accumulation in washed fruit segments. At harvest time, the I content in the I-sprayed treatments varied between 47 – 54 μg (100 g FM)^–1^ for apples and 58 – 69 μg (100 g FM)^–1^ for pears, irrespective of the addition of the aforementioned salts (Figures 4A,C). The fruit-specific differences in I enrichment leveled out again when taking into account the individual fruit weights, which in this case were higher for apples (Table 4). Without I supply – in the controls and in the stand-alone KNO_3_ foliar fertilization treatments – the I content of the fruits was about 1.0 μg (100 g FM)^–1^. During the three-monthly cold storage the I content in I-sprayed apples decreased by 20%. In the case of pears, however, fruit storage had no significant effect on the I content.

In the fruit peel of I-sprayed, washed apples and pears, the I content at harvest was 6.6 and 17.1 times higher, respectively, than in the fruit flesh. In the case of apples, this difference decreased after cold storage, as the I content in the fruit peel decreased by 45% and simultaneously increased by 14% in the fruit flesh (Table 5). For the pear, however, no significant change in this respect was observed.

**TABLE 5:** Iodine and selenium content in fruit peel and flesh of washed apples cv. ‘Fuji’ and pears cv. ‘Williams Christ’ in the second field experiment as affected by different foliar spray treatments and a fruit storage at 2°C for a period of three months.

**Apple**		**Iodine content [μg (100 g FM)^–1^]**							
		**At harvest**				**After storage**			
**Treatment**		**Fruit peel**		**Fruit flesh**		**Fruit peel**		**Fruit flesh**	
		
1	Control	2.1 ± 0.2	a A	0.5 ± 0.2	a A	2.3 ± 0.1	a A	0.5 ± 0.1	a A
2	KNO_3_	2.3 ± 0.4	a A	0.5 ± 0.4	a A	2.0 ± 0.5	a A	1.0 ± 0.5	a A
3	KIO_3_	219.2 ± 53.2	b A	26.7 ± 8.3	b A	125.3 ± 75.4	b B	29.9 ± 6.5	b A
4	KIO_3_ + KNO_3_	172.8 ± 62.7	b A	27.1 ± 3.9	b A	84.0 ± 60.0	b B	28.8 ± 10.5	b A
5	KIO_3_ + Na_2_SeO_4_ + KNO_3_	157.2 ± 79.6	b A	29.0 ± 6.9	b A	92.3 ± 49.9	b A	36.1 ± 8.9	b A
		**Selenium content [μg (100 g FM)^–1^]**							
		
1	Control	0.5 ± 0.2	a A	0.4 ± 0.1	a A	0.9 ± 0.4	a A	0.4 ± 0.2	a A
5	KIO_3_ + Na_2_SeO_4_ + KNO_3_	10.9 ± 3.2	b A	1.4 ± 0.4	b A	7.2 ± 5.3	b A	2.0 ± 0.7	b A

**Pear**		**Iodine content [μg (100 g FM)^–1^]**							
		**At harvest**				**After storage**			
**Treatment**		**Fruit peel**		**Fruit flesh**		**Fruit peel**		**Fruit flesh**	
		
1	Control	3.4 ± 0.7	a A	0.7 ± 0.7	a A	1.3 ± 0.8	a B	1.0 ± 0.8	a A
2	KNO_3_	4.8 ± 0.5	a A	0.8 ± 0.5	a A	4.1 ± 0.3	a A	0.4 ± 0.3	a B
3	KIO_3_	304.9 ± 17.4	b A	17.9 ± 3.3	b A	406.1 ± 145.9	b A	16.5 ± 5.3	b A
4	KIO_3_ + KNO_3_	356.3 ± 78.7	b A	19.7 ± 4.1	b A	366.7 ± 136.0	b A	35.2 ± 6.9	b A
5	KIO_3_ + Na_2_SeO_4_ + KNO_3_	355.0 ± 52.0	b A	22.0 ± 7.8	b A	331.8 ± 97.2	b A	21.5 ± 8.7	b A
		**Selenium content [μg (100 g FM)^–1^]**							
		
1	Control	0.4 ± 0.0	a A	0.1 ± 0.1	a A	0.5 ± 0.4	a A	0.1 ± 0.0	a A
5	KIO_3_ + Na_2_SeO_4_ + KNO_3_	4.3 ± 2.1	b A	2.0 ± 0.5	b A	6.5 ± 5.2	b A	1.6 ± 0.7	b A

*Means ± standard deviation (apple *n* = 6, pear *n* = 4). Means not sharing a lower case letter in one column or an upper case letter for same type of fruit sample in one row are significantly different according to Tukey-HSD test at α = 0.05.*

**FIGURE 5:**
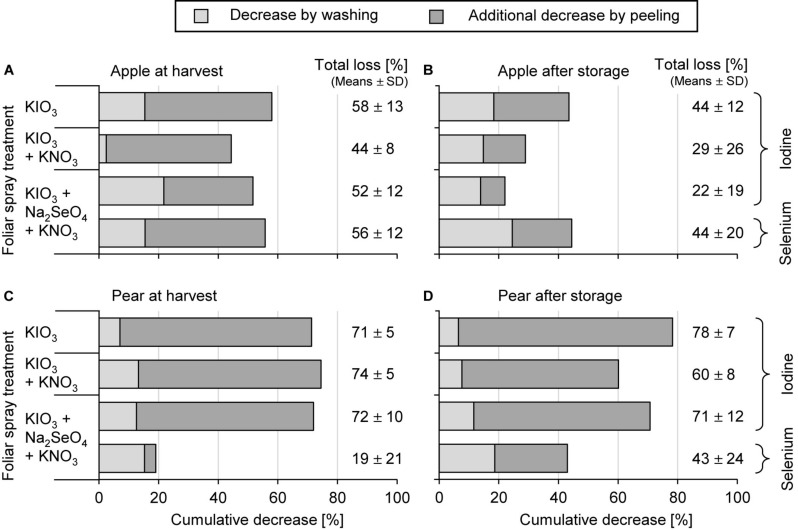
Cumulative decrease of the iodine and selenium content in fruit segments by washing and peeling of apples cv. ‘Fuji’ **(A,B)** and pears cv. ‘Williams Christ’ **(C,D)** in the second field experiment at harvest time and after fruit storage at 2°C for a period of three months. Means ± standard deviation (apple *n* = 6, pear *n* = 4).

Washing the fruit segments of I-treated apples and pears under running deionised water reduced their I content at harvest time by 13% and 11%, respectively, which is in a similar order of magnitude to that observed in the first field experiment. In peeled fruit segments the I content was reduced by 51% and 73%, respectively (Figure 5). In stored apples the I losses due to peeling were lower, as expected, due to the previously reported decrease of I content in the apple peel.

Leaves accumulated considerably more I than fruit, as exemplary analyses on apple trees revealed. Unwashed apple leaves not sprayed with I contained 166 ± 67 μg I (100 g FM)^–1^. As a result of a KIO_3_ foliar application, the I content increased to 10,924 ± 1,712 μg (100 g FM)^–1^. In washed leaves, this was at a similar level with 11,082 ± 1,778 μg (100 g FM)^–1^. The mean dry matter content of apple leaves was 37%. The aforementioned I content on fresh matter basis thus corresponds to 300 mg (kg DM)^–1^.

### Selenium Content of Fruits and Leaves

The native Se content of apples and pears was 0.4 μg (100 g FM)^–1^ and 0.1 μg (100 g FM)^–1^, respectively. Repeated foliar sprays of Na_2_SeO_4_ with a total of 50 g Se (ha ⋅ m CH)^–1^ increased the Se content in washed fruit segments to 2.7 μg (100 g FM)^–1^ and 2.1 μg (100 g FM)^–1^, respectively (Figures 4B,D). Cold storage of the fruits had no effect on the Se content.

The foliar-applied Se was enriched in the fruit peel of apples and pears by a factor of 7.8 and 2.2, respectively, more than in the fruit flesh (Table 5). Washing and peeling reduced the Se content in these pome fruits 15% and 38%, respectively (Figure 5). At harvest time these losses were lower for pears than for apples. After storage, no differences were observed in this respect.

Apple leaves of the control treatments contained 1.9 ± 0.4 μg Se (100 g FM)^–1^ in the unwashed and 1.2 ± 0.6 μg Se (100 g FM)^–1^ in the washed state. Selenium fertilization increased the Se content to 303.6 ± 65.4 μg (100 g FM)^–1^ in unwashed and 309.3 ± 57.2 μg (100 g FM)^–1^ in washed leaves.

### Phytotoxicity Symptoms on Leaves

**FIGURE 6:**
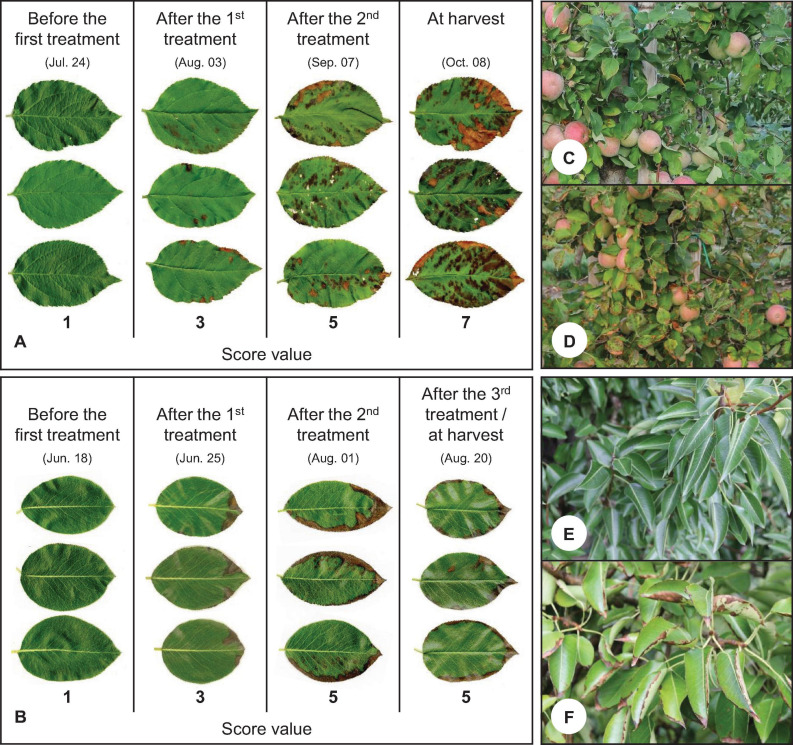
Development of leaf damage during the growing season until fruit harvest in the second field experiment. Images of scanned leaves of apple trees cv. ‘Fuji’ **(A)** and pear trees cv. ‘Williams Christ’ **(B)** from treatment no. 5 consisting of a combined foliar spray with KNO_3_, KIO_3_ and Na_2_SeO_4_. Score values indicate the degree of the damage (Score value 1 = no damage, 3 = slight damage 5 = moderate damage, 7 = severe damage, 9 = very severe damage). Detail view of ‘Fuji’ apple trees **(C,D)** and ‘Williams Christ’ pear trees **(E,F)** in the second field experiment at harvest time. Picture **C** and **E**: treatment no. 1 (control). Picture **D** and **F**: treatment no. 5 (spray solution composition as described above).

**FIGURE 7:**
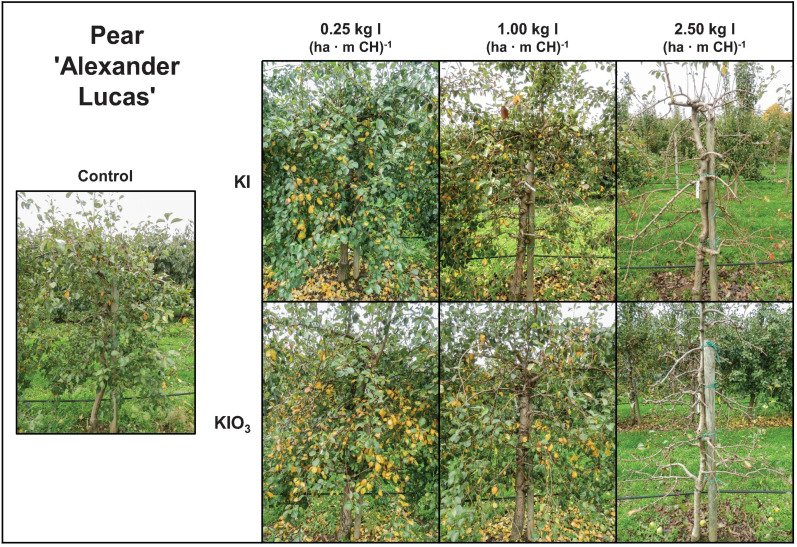
Appearance of pear trees cv. ‘Alexander Lucas’ in the first field experiment 19 days after harvest (Oct. 13) as affected by the dose and form of iodine foliar sprays applied two weeks before fruit harvest.

The spraying of I-containing solutions on apple and pear trees resulted in leaf necrosis, starting at the leaf margins and at the leaf tip. The intensity of these symptoms increased as the number of applications increased and the growing season progressed (Figure 6). In the first field trial, the leaves of pear trees showed more severe damage, while in the second field trial the leaves of apple trees were more affected (Table 6). The degree of damage increased with increasing concentration of I in the spray solution. Iodine fertilizer form had no consistent influence on the leaf damage. Likewise, the co-application of KNO_3_ and Na_2_SeO_4_ with I had no effect on damage pattern. When only KNO_3_ was sprayed, the leaves remained undamaged as in the controls. After harvesting, accelerated leaf senescence and premature leaf fall was observed in the I-sprayed treatments. These effects also increased with increasing I supply (Figure 7).

**TABLE 6:** Score values of leaf damage on trees of apples cv. ‘Jonagold’ and ‘Fuji’ and pears cv. ‘Alexander Lucas’ and ‘Williams Christ’ from the first and the second field experiment as affected by the spray solution.

**Treatment^1)^**				**Score values of leaf damage [1–9]**	
				**Apple**	**Pear**
**First field trial**	1	Control	0	1.0 ± 0.0	1.0 ± 0.0
	
	2	KI	0.25	3.0 ± 0.0	5.0 ± 0.0
	3		1.0	3.2 ± 0.5	7.2 ± 0.5
	4		2.5	5.7 ± 1.5	7.7 ± 1.0
	
	5	KIO_3_	0.25	2.4 ± 0.6	5.0 ± 0.0
	6		1.0	3.7 ± 1.0	6.5 ± 1.0
	7		2.5	6.7 ± 1.5	9.0 ± 0.0

**Second field trial**	1	Control	0	1.0 ± 0.0	1.0 ± 0.0
	
	2	KNO_3_	0	1.0 ± 0.0	1.0 ± 0.0
	3	KIO_3_	1.5	6.0 ± 1.1	3.5 ± 1.0
	4	KIO_3_ + KNO_3_	1.5	5.7 ± 1.0	4.0 ± 1.2
	5	KIO_3_ + Na_2_SeO_4_ + KNO_3_	1.5	5.7 ± 1.0	4.0 ± 1.2

*Score value 1 = no damage, 3 = slight damage 5 = moderate damage, 7 = severe damage, 9 = very severe damage. Means ± standard deviation (*n* = 4, except ‘Fuji’ apples second field trial *n* = 6). ^1)^ Iodine application rate expressed in kg (ha ⋅ m CH)^–1^.*

### Fruit Development and Content of Total Soluble Solids

**FIGURE 8:**
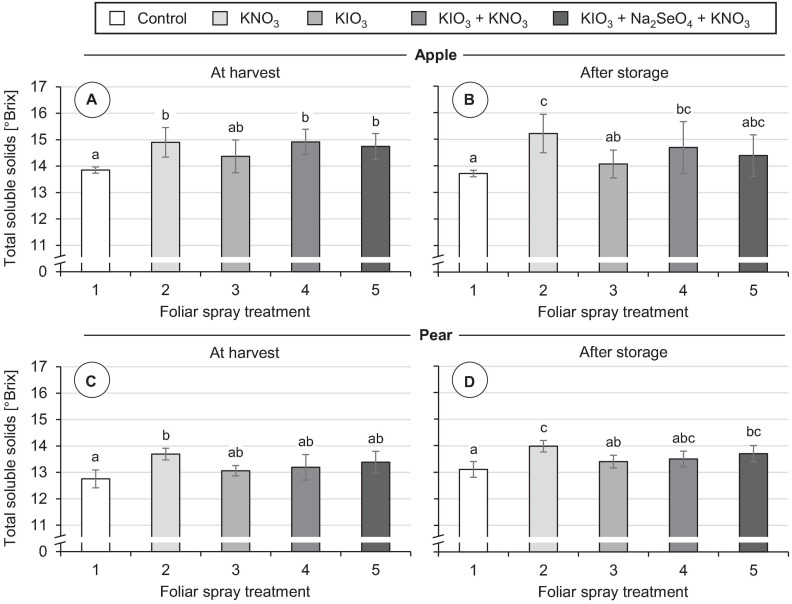
Total soluble solid content in fruit segments of apples cv. ‘Fuji’ **(A,B)** and pears cv. ‘Williams Christ’ **(C,D)** in the second field experiment as affected by different foliar spray treatments and fruit storage at 2°C for a period of three months. Means ± standard deviation (apple *n* = 6, pear *n* = 4). Means with same letters for one fruit group and one time of measurement do not differ according to Tukey-HSD test at α = 0.05.

No damage was observed on the fruits in any of the foliar fertilization treatments tested, neither at the time of harvest nor after storage. In all treatments the individual fruit weight was at the same level as in the controls (Table 4). KIO_3_ sprays did not affect the total soluble solids content of fruits. However, repeated applications of KNO_3_ promoted the accumulation of soluble solids. At harvest time the concentration of soluble solids was increased by 1.0 °Brix in apples and 0.9 °Brix in pears compared to the control (Figure 8). Even with simultaneous application of KIO_3_ and KNO_3_, apples still showed a correspondingly increased °Brix value. After cold storage of the fruits the above-mentioned differences in total soluble solids content remained.

## Discussion

### Biofortification With Straight Iodine Foliar Sprays

By applying I-containing foliar fertilizers in an orchard, it was possible to enrich apples and pears significantly with I. While washed fruit segments of the control treatments had an I content of ≤ 1.5 μg (100 g FM)^–1^, this was increased by a factor of 10 – 193 in the I-fertilized treatments and reached more than 200 μg (100 g FM)^–1^ at the highest supply rate [2.5 kg I (ha ⋅ m CH)^–1^] for both fruit species. A linear relationship between I fertilizer amount and I fruit enrichment was observed (Figure 2). The increase was 2.5 times higher with I^–^-supply than with IO_3_^–^-supply. Similar effects on the influence of the level and form of I supply were described in studies on the biofortification of strawberries (Li et al., 2017) and various vegetable and cereal crops (Hong et al., 2008; Voogt et al., 2010; Cakmak et al., 2017; Li et al., 2018).

The higher accumulation of exogenously applied I^–^ in plants is probably due to its smaller molecular weight and lower valence compared to IO_3_^–^ (Mackowiak and Grossl, 1999). Furthermore, studies on I uptake by roots indicate that IO_3_^–^ enters the symplast only after reduction to I^–^ (Kato et al., 2013). Iodide is absorbed via ion channels or chloride transporters driven by proton pumps (White and Broadley, 2009; Medrano-Macías et al., 2016). For foliar sprays, however, another aspect is probably of great importance, namely the difference in the point of deliquescence (POD) of the applied salts. The POD describes the relative humidity (RH) at which hygroscopic salts absorb enough water from the air to form a liquid solution. At a temperature of 20°C, the POD for KI is 69% RH and for KIO_3_ 93% RH (Greenspan, 1977; Apelblat and Korin, 1998). This difference will influence the capacity of spray drop deposits to rehydrated under high RH conditions as affected by temperature and hence favor new cycles of I absorption by the fruits and the foliage. KI is superior to KIO_3_ in this respect because it deliquesces at much lower RH. The deliquescence phenomena will be more prone to occur at night time and also in association with dew on plant organs. In our field experiments spray applications were always carried out in the morning hours when RH was usually below 94% and the thin spray liquid films formed on the surface of fruits and leaves dried relatively quickly. As a result, the dissolved salt can be converted to the solid, crystalline state. The RH at which this phase transformation begins is defined as the point of efflorescence (POE). The POE is usually below that of the POD (Freney et al., 2009). Recently, the importance of the POE of fertilizer salts for spray drop drying has been highlighted (Fernández et al., 2020). To the best of our knowledge, POE values for KI and KIO_3_ have not yet been published and thus should be determined in further investigations. The translocation of I from the fruit peel to the fruit flesh was also affected by the I form as well as by the type of fruit. In the case of IO_3_^–^ supply, 14% and 44% of the I were found in the fruit flesh of the pear and apple, respectively, while in the case of I^–^ supply the figures were 27% and 51%, respectively (Figure 3).

Despite the higher absorption and translocation of I^–^, the I content in single pome fruits was subject to greater fluctuations within the treatments compared to IO_3_^–^, especially at high I supply. Likewise, in other published field experiments IO_3_^–^ led to a more uniform result when applied at different locations and under varying environmental conditions (Lawson et al., 2016; Cakmak et al., 2017). This is advantageous for the practical implementation of agronomic biofortification, where the aim is to achieve the desired level of I biofortification in a way that is as reproducible as possible. For this reason, we selected KIO_3_ as I salt for our second field experiment. With an application rate of 1.5 kg I (ha ⋅ CH)^–1^ it was possible to increase the I content in washed fruit segments to about 50–60 μg (100 g FM)^–1^ (Figure 4). An I enrichment of the same order of magnitude was achieved when apple trees cultivated under protected conditions in a plastic tunnel were sprayed with I (Budke et al., 2020a). This is surprising, since in an orchard it can be expected that exogenously applied I will be partially washed off the fruit trees by rain. In the second field experiment, the amount of precipitation that fell in the period from the first foliar spray to the harvest of fruits was 78.2 mm (over 75 days) for apples and 100 mm (over 63 days) for pears. However, no or very low precipitation (< 3 mm) was observed in the first two days after application. In the first field experiment, however, about 6 mm of precipitation fell one day after the treatment of the apple trees. Nevertheless, the apples investigated here were also enriched with I to an extent similar to the described plastic tunnel experiment. Obviously, rainfall in the range mentioned did not result in noteworthy wash-off losses even if the I sprayed on the fruit was probably not absorbed completely within one day. Investigations on butterhead lettuce showed that after one day only about half of the I deposited on leaves via foliar fertilization was absorbed by the leaves (Lawson et al., 2016). In fruits, especially those with a thicker wax layer on the surface, the uptake of I is likely to proceed much more slowly, although this has not yet been investigated. Studies on calcium uptake in apples of the ‘Cox Orange’ variety showed that within 2 to 7 days a maximum of 7% and 25%, respectively, of the radioisotope ^45^Ca^2+^ applied to the fruit surface penetrated to a depth of 1 mm into the fruit (van Goor, 1973).

When evaluating I biofortification of apples and pears, fruit size must be taken into account. As the fruit weight increases, the I absorbed into the fruit becomes increasingly diluted (Budke et al., 2020a). Accordingly, a higher I fertilizer application was required for bigger pears of the ‘Alexander Lucas’ variety in order to achieve an I content comparable to that of the smaller ‘Williams Christ’ pears. With regard to the total amount of I contained in the pears, only minor differences between both pear varieties were found. At a KIO_3_ application rate of 1.5 kg I (ha ⋅ CH)^–1^, pears of the ‘Williams Christ’ variety still contained about 10% more I than determined for ‘Alexander Lucas’ by calculation (Table 4).

The apple varieties ‘Fuji’ and ‘Jonagold’ hardly differed in fruit size and showed a similar I accumulation patterns in the fruits at the same KIO_3_ application rate. The thickness of the epicuticular wax layer of the two apple varieties is also comparable and is in the middle to higher range for apples at harvest time with approx. 1.5 μm. In general, the wax deposition on the apple peel increases as the fruit develops (Guan et al., 2015). Therefore, a late foliar application date, as set in the first field experiment with the variety ‘Jonagold’ (treatment two weeks before harvest), would rather result in a lower uptake rate of I sprayed on the fruit. On the other hand, the surface area of growing fruits increases during the season. Thus, more I is retained by the fruit if the application date is late. Taken together it can be assumed that these two opposing effects compensated each other and therefore the different treatment dates in the field experiments performed had no influence on fruit I accumulation.

Preferential uptake routes for dissolved ionic solutes into the fruit are fine cracks in the cuticle and lenticels (Harker and Ferguson, 1988). The occurrence of these epidermal structures can vary considerably depending on the variety. ‘Williams Christ’ pears, for example, have more than three times as many lenticels as ‘Alexander Lucas’ pears (Durić et al., 2015). In our experiments, this may have additionally favored the I enrichment in the smaller fruiting ‘Williams Christ’ variety. ‘Fuji’ apples are known to have significantly more lenticels on the fruit surface than ‘Jonagold’ apples (Guan et al., 2015). However, in contrast to pears, these differences in variety did not affect the I uptake of apples. Thus, from our data we cannot conclude that lenticels play an important role for I fruit absorption.

Even though foliar sprays with I-containing fertilizers have proven to be suitable for production of biofortified pome fruits with increased I content, the efficiency of this measure is relatively low. In a normal orchard with a tree height of 2.5–3.0 m and a fruit yield of 40 t ha^–1^, no more than about 0.5% of the applied I enters the fruits if their I content averages 50 μg (100 g FM)^–1^. This calculation is based on a fertilization of 1.5 kg I (ha ⋅ m CH)^–1^ in the form of KIO_3_. When using KI, the proportion of I transferred into the fruits can increase up to 1.1%, since a lower amount of I fertilizer is required for the same I enrichment.

It may be possible to increase the efficiency of I foliar fertilization by using an air-blast orchard sprayer, which is commonly used in commercial fruit growing. This application technique is likely to be superior to the handheld sprayers used in the experiment, especially with regard to sufficient wetting of the fruits covered by leaves inside the tree. This is important because they must be hit directly by the spray solution in order to be significantly biofortified. The translocation of I from leaves to fruits in apple trees was found to be negligible, which is attributed to a low phloem mobility of I in apple trees (Budke et al., 2020a). Most of the I applied by foliar fertilization is probably found in the foliage, which has a surface area more than 10 times larger than the fruits growing on the tree (Knoche and Petracek, 2014). The I content measured in apple leaves was more than 200 times higher than the fruits. The main reason for this is certainly the larger surface area-to-volume ratio of the leaves, which means that the increase in concentration is higher for the same amount of solutes per unit of area. In addition, the epidermis of the leaves is covered by a thinner wax layer than that of the fruits (Fernandes et al., 1964) and stomata are available as additional uptake routes for ionic solutes (Eichert and Fernández, 2012).

### Biofortification With Leaf Fertilizer Mixtures

The addition of KNO_3_ to a spray solution containing KIO_3_ had no effect on the I content of the fruits, neither for apple nor for pear (Figure 4). In contrast, Cakmak et al. (2017) found in a study on wheat plants that the uptake of foliar-applied IO_3_^–^ is significantly increased by KNO_3_. It is not yet clear what this positive effect was due to. An effect as humectant is not considered here, since KNO_3_ has a relatively high deliquescence point with 95% RH (Fernández et al., 2013). Stronger hygroscopic salts such as CaCl_2_ (deliquescence point of 33% RH), on the other hand, can fulfill this purpose and thus promote I uptake into the plant tissue (Lawson et al., 2016). Further investigations must reveal whether such tank mixtures are also useful for the I fertilization of fruit crops.

The addition of Na_2_SeO_4_ to a spray solution containing IO_3_^–^ did not affect the I content of the treated pears and apples. This confirms results from previous studies on apple trees (Budke et al., 2020a). Likewise, in studies on the biofortification of lettuce and rice, no interactions between IO_3_^–^ and SeO_4_^2–^ were found with regard to the uptake of both trace elements (Smoleń et al., 2014, 2016b; Prom-u-thai et al., 2020). In contrast, in field experiments with carrots and wheat, a slight reduction of I accumulation in the edible plant parts was observed when Se was simultaneously applied to the soil or Se and other micronutrients to the leaf (Smoleń et al., 2016a; Zou et al., 2019). However, the effects were not consistent, but varied depending on year and location.

The combined foliar fertilization of KIO_3_, KNO_3_ and Na_2_SeO_4_ increased the Se content in the fruits 6 times compared to the control in apples and 21 times in pears. However, the maximum accumulation remained below 3.0 μg Se (100 g FM)^–1^ and was thus of a similar order of magnitude as previously determined for apples with a combined KI and Na_2_SeO_3_ foliar spray (Budke et al., 2020a). In both studies, the total Se fertilization rate applied was 50 g (ha ⋅ m CH)^–1^. From a human nutritional point of view, the optimal molar I/Se ratio in foods is about 6:1 (Lyons, 2018). For example, at a content of 50 μg I (100 g FM)^–1^, the target value for Se would be 5.2 μg (100 g FM)^–1^. In a study by Groth et al. (2020), Se content of this level was achieved in apples by a foliar spray of 150 g Se (ha ⋅ m CH)^–1^, regardless of whether SeO_3_^2–^ or SeO_4_^2–^ was applied. Further field experiments are needed to examine the effects of appropriately increased Se fertilization rate in combination with I. In the leaves of the apple trees we examined, the Se content was several times higher than in the fruits, as already observed with I. Translocation of I and Se from leaves to seeds in wheat is mainly through phloem transport (Cakmak et al., 2017; Prom-u-thai et al., 2020), while our findings indicate that this route does not seem important for biofortification of pome fruits.

### Effects of Fruit Storage

Cold storage of I-sprayed apples at 2°C for three months reduced the I content of the fruit by about one fifth. In contrast, no statistically significant changes were observed in pears (Figure 4). In the apples, the storage-related reduction of the I content was limited to the fruit peel, while the content in the fruit flesh remained relatively stable (Table 5). At harvest time, the I content in the fruit peel was 6.6 times higher than in the fruit flesh. However, after storage this difference was reduced to about half.

In I-biofortified nectarines, which were stored at 5°C for two weeks, the I content also remained unchanged (Caffagni et al., 2012). Gaseous emissions associated with the activity of methyltransferases have been detected in numerous plant species. These enzymes catalyze the formation of methyl iodide (CH_3_I), a volatile compound, which can escape into the atmosphere (Itoh et al., 2009). Besides a role in plant defense, this mechanism may serve to prevent toxic levels of I accumulation in higher plants (Gonzali et al., 2017). Additionally, I volatilization can be catalyzed by vanadium-dependent haloperoxidase, leading to synthesis of volatile hydrogen halides. Recently, activity of these enzymes in relation to I uptake has been demonstrated for lettuce (Smoleń et al., 2020). In brown alga *Laminaria digitata* volatilization of cellular I by vanadium-dependent haloperoxidases is thought to be a potential tool in defense against pathogens and I volatilization is important to maintain osmotic balance (Verhaeghe et al., 2008). However, to the best of our knowledge the activity of I-specific halide methyltransferases or haloperoxidase in pome fruits has not been studied.

Fruit storage did not affect the Se content of apples or pears (Figure 4). Nevertheless, it is known that plants are able to form volatile Se compounds such as dimethyl selenide [(CH_3_)_2_Se] and dimethyl diselenide [(CH_3_)_2_Se_2_] from Se-containing amino acids (Malagoli et al., 2015). However, these processes obviously do not play a significant role in stored pome fruit at the Se enrichment level achieved in our study. To what extent a longer storage time – apples can be stored under controlled atmosphere conditions until the following year’s harvest – affects fruit Se and I losses should be examined in further trials.

### Effects of Fruit Washing, Peeling and Core Removal

When fruit segments of I-biofortified apples and pears were washed under running deionised water shortly after harvesting, this reduced their I content by 14%. Losses of a similar magnitude were observed for Se in the Se-fertilized treatment (Figure 5). This shows that most of the I and Se detected in the fruits was completely absorbed or adhered so firmly to the fruit peel that it could not be removed by normal washing procedures. A strong sorption of foliar-applied I to cuticular waxes was observed on leaves of field beans (Shaw et al., 2007). Losses of I of up to 30% were observed when washing strawberries that received a final I spray six days before harvest. Longer pre-harvest intervals reduced I losses to below 20% (Budke et al., 2020b). In our experiments the pre-harvest interval was at least two weeks and in the second field trial with apples the last foliar fertilization was carried out almost six weeks before harvest.

After I foliar sprays, the fruit peel contains much higher concentrations of I compared to the flesh. Therefore, peeling lowers the I content of the fruit. In freshly harvested apples of the I-fertilized treatments, it decreased by 51% and in pears by as much as 78%. Similarly, high peeling-related I losses were previously reported for apples (Budke et al., 2020a). For nectarines, however, the peeling of I-biofortified fruits did not lead to a significant change in the I content (Caffagni et al., 2012). This may be due to the differences in fruit peel properties between pome and stone fruits affecting the penetration of I into the fruit. Furthermore, it should be noted that the I enrichment in the nectarines was lower by more than a factor of 10 compared to pome fruits that we investigated. For Se the peeling effects were subject to stronger fluctuations, which is probably due to the relatively low Se content of Se-sprayed fruits. In general, peeling of pome fruit is not recommended, since not only are larger amounts of biofortified I and Se lost but also health-promoting secondary plant compounds from the group of flavonoids, which are mainly localized in the fruit peel (Drogoudi et al., 2008).

The fruit core of I-fertilized apples and pears always had the lowest I content within the fruit [5.6 μg (100 g FM)^–1^]. In total, not more than 1 – 3% of the I contained in a fruit was found in the fruit core. This indicates that only a small part of the I absorbed via the fruit surface penetrated to the center of the fruit.

As the core of apples and pears is usually not consumed, the limited translocation of the I in the fruit is advantageous with regard to its utilization for human nutrition. The I content in fruits without the core was about 9% higher in apples and about 14% higher in pears than in the whole fruit. This difference must be taken into account when in future the I content needs to be determined for quality control procedures and the marketing of I-biofortified pome fruit. In this case, it is useful to analyze the I content in washed, cored fruits in order to indicate adequately the contribution of the products to the dietary I intake.

### Content of Total Soluble Solids

The total soluble solids content is often used as an indicator for the sugar content and sweetness of fruits (Charles et al., 2017). These fruit characteristics have a significant influence on the taste and consumer acceptance of apples and pears (Hoehn et al., 2003; Predieri et al., 2014). Spraying KIO_3_ alone did not lead to a significant change in total soluble solids content in either of the two pome fruits analyzed. In apples, a combined application of KIO_3_ and KNO_3_ increased the total soluble solids content by about 1.0 °Brix. An increase of the same order occurred in apples as well as in pears when a pure KNO_3_ leaf fertilizer was applied. These positive effects remained even after three months of cold storage of the fruits (Figure 8). In accordance with this, Shen et al. (2016) report that foliar sprays with KNO_3_ in ‘Kousui’ Japanese pears led to an increase of fructose and sucrose content in the fruits and thereby significantly increased their sweetness. Other potassium-containing fertilizers also had a beneficial effect in this respect. Potassium plays an important role in the photosynthesis of the leaves and the translocation of the assimilates into the fruits (Zörb et al., 2014). Nevertheless, the positive influence of potassium foliar fertilization on the sugar content of the fruits in our field experiment is surprising, since the plant-available potassium content of the soil at the experimental site was in the optimal range [class C according to VDLUFA (Kießling and Hoffmann, 2016)]. The effect of I on sugar accumulation in fruits can vary considerably depending on the amount of I applied, as shown by studies on strawberries. In hydroponically cultivated strawberries, a moderate increase of the I concentration in the nutrient solution promoted the accumulation of soluble sugars in fruits. In contrast, high I concentrations in the nutrient solution reduced the fruit sugar content (Li et al., 2017). Likewise, after repeated KI sprays on strawberries grown in the field, a significant reduction of total soluble solids content was observed when a total of 0.8 kg I ha^–1^ was applied. In contrast, I fertilizer applications of ≤ 0.4 kg I ha^–1^ had no such adverse effects (Budke et al., 2020b).

The addition of Na_2_SeO_4_ in fertilizer mixtures with KIO_3_ and KNO_3_ did not affect the total soluble solids content of apples and pears. Pezzarossa et al. (2012), however, reported that pure Se spraying of pear trees led to a significant increase in the total soluble solids content of fruits. In this field experiment Na_2_SeO_4_ was also used, but with a significantly lower concentration in the spray solution (1.0 mg Se L^–1^) than in our study (50 mg Se L^–1^). In peaches, which were also included in the aforementioned study, no corresponding effects were found depending on Se fertilization (Pezzarossa et al., 2012). In hydroponically cultivated strawberries, it was possible to increase the total soluble solids content by about 2.0 °Brix if about 8 mg Se L^–1^ was added to the nutrient solution as Na_2_SeO_4_ (Mimmo et al., 2017). In grapes, the content of glucose, fructose and sucrose correlated closely with the Se content of the fruits. Here, Se was added by application of a leaf fertilizer containing 120 mg L^–1^ organically bound Se in the spray solution (Zhu et al., 2017).

Taken together, it appears that, in addition to potassium, I and Se can also promote the accumulation of sugar in fruits. However, there are differences in this respect depending on the type of fruit, the fertilization level, the form of fertilization and probably also the application technique, which need to be further investigated.

### Phytotoxic Effects

Spraying with I-containing fertilizers caused leaf necroses on apple and pear trees, which increased with increasing I doses (Figures 6, 7). Similar damage was previously observed in different plant species (Caffagni et al., 2011; Kato et al., 2013; Kiferle et al., 2013; Cakmak et al., 2017; Incrocci et al., 2019; Budke et al., 2020a,b). At equal concentrations I^–^ usually causes stronger phytotoxic effects than IO_3_^–^. One reason for this might be that I^–^ inhibits the activity of superoxide dismutase, while IO_3_^–^ can promote its activity. This enzyme plays a key role in the defense against reactive oxygen species and thus in the prevention of cell damage (Blasco et al., 2011). In our study, however, no consistent differences between the two I species were observed with respect to the intensity of leaf damage.

The fruits of the apple and pear trees did not experience any sort of damage (Figure 1), even after three months of cold storage. The individual fruit weight also remained unaffected (Table 4). Furthermore, as discussed before, the total soluble solids content of the fruits was not reduced by I applications, and in combination with KNO_3_ even increased significantly in some cases. Thus, we assume that the observed leaf damage had no negative influence on the fruit development. In the year after application, no abnormalities, e.g., with regard to fruit set or fruit development, were observed on the I- and Se-fertilized trees in the experiments conducted as well as in other investigations not yet published. However, we cannot exclude the possibility that long-term I and Se supply have an adverse effect on fruit trees. To clarify this, fertilization trials in orchards over several years are necessary.

### Implementation of Iodine Biofortification in Pome Fruit Production

The biofortification of pome fruit with I can be integrated into fruit growing practice by means of foliar fertilization with relatively little effort and at acceptable costs. The application can be done with a standard orchard sprayer. With a raw material price of 60 US-$ per kg KIO_3_ in food grade, an exchange rate of 1.18 US-$ per € and a fertilization quantity of 1.5 kg I (ha ⋅ m CH)^–1^, the pure I fertilizer costs in an orchard with 2.5 – 3.0 m high trees amount to about 320 – 385 € ha^–1^. In addition, there are the application costs, which are estimated to be about 50 € ha^–1^ per treatment (Weitgruber, 2016). Overall, with an average yield of 40 t, the I biofortification would result in additional costs of around 1.0 – 1.3 euro cents per kg of fruit. In the case of apple cultivation, for example, this would correspond to about 2.5 – 3.5 % of total production costs (Lang and Thomann, 2008). The application costs are omitted or arise only proportionately if the I treatment can be combined with other sprays. KNO_3_ and Na_2_SeO_3_ have proved to be suitable mixture components in the concentrations tested in our experiments.

Repeated calcium sprays are common in pome fruit cultivation, among other things to prevent physiological disorders such as bitter pit in apples or flesh browning in pears (Blanco et al., 2010; Wójcik, 2012). Therefore, further investigations should be carried out to determine whether I can also be applied together with this plant nutrient. However, when using IO_3_^–^ as I species, miscibility is limited here by the low water solubility of Ca(IO_3_)_2_, which is 2.43 g L^–1^ at 20°C (John, 2019). In 600 liters of water, which are usually applied with an orchard sprayer per hectare, up to 0.95 kg I could be dissolved as Ca(IO_3_)_2_. Thus, for the application of 3.75 – 4.50 kg IO_3_^–^-I ha^–1^, four to five treatments with such a spray solution are necessary. If I^–^ is used, the required I supply can be achieved with fewer treatments, since CaI_2_ is much more soluble in water [676 g L^–1^ at 20°C (John, 2019)]. In field experiments with lettuce, the addition of calcium nitrate [Ca(NO_3_)_2_] to an IO_3_^–^-containing spray solution had no effect on I uptake into the foliage, while CaCl_2_ was beneficial (Lawson et al., 2016). Tank mixtures of KIO_3_ with selected pesticides were also successfully tested in the aforementioned study.

It was also possible to achieve the I enrichment targeted for apples and pears by a single foliar fertilization with KIO_3_ or KI (Figure 2). In our first field experiment, this treatment was applied two weeks before harvest. At the highest fertilization level, with 2.5 kg I (ha ⋅ m CH)^–1^, the trees were largely defoliated three weeks after harvest (Figure 7). This conspicuous side effect of I sprays could possibly be used in pome fruit cultivation to promote the coloration of the fruits, especially of varieties with red peel color, by improving exposure to light. In further investigations it will be necessary to check which treatment date and which I application quantities are particularly suitable for this purpose. Currently, a technique is being tested for pre-harvest defoliation of apple trees in which the outer leaves are removed by means of compressed air two to four weeks before harvesting (Andergassen and Pichler, 2019). This requires first of all the purchase of a special defoliation machine. Furthermore, it should be noted that the pneumatic defoliation can lead to increased fruit drop and pressure marks on the fruit. Last but not least, the associated treatment costs of around 1,600 € ha^–1^ (Andergassen and Pichler, 2019) are significantly higher than for I sprays.

A premature leaf fall in apple trees could also be interesting from a phytosanitary point of view. The ascospores of the apple scab (*Venturia inaequalis*), from which the primary infection starts in spring, overwinter on the fallen leaves. In order to ensure a rapid conversion of the leaf material, urea sprayings are carried out after harvesting and the fallen leaves are then mulched (Holb, 2006; Singh, 2019). The earlier this is done in autumn, the more complete the decomposition process can progress. To what extent a late I application is useful in this respect and whether such a treatment can contribute to the reduction of scab infestation in an apple orchard should be investigated in further field trials.

## Conclusion

Pome fruits can be biofortified with I to an extent appropriate for human nutrition when cultivated under orchard conditions by means of foliar fertilizer sprays. The supply of KIO_3_ at a total application rate of 1.5 kg I (ha ⋅ m CH)^–1^ increased the I content in washed apples and pears to about 50 – 60 μg (100 g FM)^–1^ without affecting the development and marketability of the fruits. The consumption of such an I-enriched fruit of average size (about 175 g) would cover about two thirds of the recommended daily I intake of 150 μg for an adult (EFSA, 2006). Foods declared and marketed in the European Union with nutritional claims must have a certain I content in accordance with Regulation (EC) No 1924/2006 (European Commission, 2011). With an I content of ≥ 22.5 μg (100 g FM)^–1^, corresponding to 15 % of the recommended daily allowance for I, foods may be labeled as a “source of iodine.” If the I content is twice as high, the products can be labeled as “rich in iodine.” Such foods may also be advertised with health claims such as “iodine contributes to normal thyroid function” according to Regulation (EU) No. 432/2012 (European Commission, 2012). The approach thus offers fruit producers an interesting option for increasing the nutritional value of their products, and to take advantage of this in marketing.

In the field experiments performed, only fruits hanging on the outside of the tree and thus those directly wetted by the spray solution were examined. It can be assumed that fruits from inside the tree, which were partially or entirely covered by leaves, had lower I contents. Therefore, in further investigations variations in the range of the I enrichment of the fruits depending on their position on the tree should be investigated. In this context, the application technique used might also play an important role. With air-blast orchard sprayers, as used in commercial tree fruit cultivation, a significantly better penetration can probably be achieved than with the hand sprayers and backpack sprayers used in our experiments. With regard to the practical use of I biofortification in pome fruit cultivation, it also remains to be clarified what influence foliar fertilizer additives such as adhesive agents as well as tank mixtures with calcium-containing fertilizers and pesticides have on the effectiveness of the process. Furthermore, it is important to discover how fast the sprayed I penetrates the fruit and to what extent weather conditions affect this.

## Data Availability Statement

The raw data supporting the conclusions of this article will be made available by the authors, without undue reservation.

## Author Contributions

CB and DD conceived and designed the field experiments. CB conducted the field experiments and analytical investigations, analyzed the data, and wrote the manuscript, together with DD. DD and KM supervised the analytical investigations. DD, KH, KM, and WD provided resources to conduct the field experiments and analytical investigations. H-GS supervised the statistical data analysis. All authors contributed to manuscript revision, reading, and approved the submitted version.

## Conflict of Interest

KH is an employee of SQM INTERNATIONAL N.V., a company active in the sector of fertilizers. The remaining authors declare that the research was conducted in the absence of any commercial or financial relationships that could be construed as a potential conflict of interest.

## Abbreviations

CH: canopy height
DM: dry matter
FM: fresh matter
GF-AAS: graphite furnace atomic absorption spectrometry
ICP-MS: inductively coupled plasma mass spectrometry
POD: point of deliquescence
POE: point of efflorescence
TMAH: tetramethylammonium hydroxide
VDLUFA: Association of German Agricultural Analytic and Research Institutes.
