# Supplementary material for: Iodine Biofortification of Apples and Pears in an Orchard Using Foliar Sprays of Different Composition
Source: Front Plant Sci. 2021 Feb 24;12:638671. doi: 10.3389/fpls.2021.638671 (PMC7943743; doi:10.3389/fpls.2021.638671)
Supplement: Supplementary file 1 [file Data_Sheet_1.PDF]

Tab. SM1 | Log-transformed iodine content in washed fruit segments of apples cv. 'Jonagold' at harvest time as affected by the dose and form of iodine foliar sprays in the first field experiment (*n* = 4).

Foliar spray treatment [kg I (ha · m CH)$^{-1}$]	KI	KIO_3	Mean Doses
0.00	0.354	0.343	0.349
0.25	1.243	1.184	1.213
1.00	1.951	1.554	1.753
2.50	2.436	1.989	2.213
Mean Forms	1.496	1.268	
Analysis of Variance			
Forms (F)	***		
Doses (D)	***		
F x D	***		
LSD 5% Forms	0.059		
LSD 5% Doses	0.084		
LSD 5% F x D	0.118		

Tab. SM2 | Log-transformed iodine content in washed fruit segments of pears cv. 'Alexander Lucas' at harvest time as affected by the dose and form of iodine foliar sprays in the first field experiment (n = 4).

Foliar spray treatment [kg I (ha · m CH)$^{-1}$]	KI	KIO_3	Mean Doses
0.00	0.084	0.084	0.084
0.25	1.165	1.126	1.145
1.00	1.936	1.463	1.699
2.50	2.335	1.716	2.026
Mean Forms	1.380	1.097	
Analysis of Variance			
Forms (F)	***		
Doses (D)	***		
F x D	**		
LSD 5% Forms	0.112		
LSD 5% Doses	0.158		
LSD 5% F x D	0.224		
